# Using Serum α-Fetoprotein for Prognostic Prediction in Patients with Hepatocellular Carcinoma: What is the Most Optimal Cutoff?

**DOI:** 10.1371/journal.pone.0118825

**Published:** 2015-03-04

**Authors:** Chia-Yang Hsu, Po-Hong Liu, Yun-Hsuan Lee, Cheng-Yuan Hsia, Yi-Hsiang Huang, Han-Chieh Lin, Yi-You Chiou, Fa-Yauh Lee, Teh-Ia Huo

**Affiliations:** 1 Faculty of Medicine, National Yang-Ming University School of Medicine, Taipei, Taiwan; 2 Institute of Clinical Medicine, National Yang-Ming University School of Medicine, Taipei, Taiwan; 3 Institute of Pharmacology, National Yang-Ming University School of Medicine, Taipei, Taiwan; 4 Department of Medicine, Taipei Veterans General Hospital, Taipei, Taiwan; 5 Department of Surgery, Taipei Veterans General Hospital, Taipei, Taiwan; 6 Department of Radiology, Taipei Veterans General Hospital, Taipei, Taiwan; 7 Department of Biostatistics, University of California Los Angeles, Los Angeles, California, United States of America; National Cancer Institute, UNITED STATES

## Abstract

**Background and Aims:**

The prognostic ability of α-fetoprotein (AFP) for patients with hepatocellular carcinoma (HCC) was examined by using different cutoff values. The optimal AFP cutoff level is still unclear.

**Methods:**

A total of 2579 HCC patients were consecutively enrolled in Taiwan, where hepatitis B is the major etiology of chronic liver disease. Four frequently used AFP cutoff levels, 20, 200, 400, 1000 ng/mL, were investigated. One-to-one matched pairs between patients having AFP higher and lower than the cutoffs were selected by using the propensity model. The adjusted hazard ratios of survival difference were calculated with Cox proportional hazards model.

**Results:**

Patients with a higher AFP level were associated with more severe cirrhosis, more frequent vascular invasion, higher tumor burden and poorer performance status (all p<0.0001). In the propensity model, 4 groups of paired patients were selected, and there was no difference found in the comparison of baseline characteristics (all p>0.05). Patients with AFP <20 ng/mL had significantly better long-term survival than patients with AFP ≧20 ng/mL (p<0.0001), and patients with AFP <400 ng/mL had significantly better overall outcome than patients with AFP ≧400 ng/mL (p = 0.0186). There was no difference of long-term survival between patients divided by AFP levels of 200 and 1000 ng/mL. The adjusted hazard ratios of AFP ≧20 ng/mL and AFP ≧400 ng/mL were 1.545 and 1.471 (95% confidence interval: 1.3–1.838 and 1.178–1.837), respectively.

**Conclusions:**

This study shows the independently predictive ability of baseline serum AFP level in HCC patients. AFP levels of 20 and 400 ng/mL are considered feasible cutoffs to predict long-term outcome in unselected HCC patients.

## Introduction

Hepatocellular carcinoma (HCC) is one of the most common malignant neoplasms worldwide [1,2]. α-fetoprotein (AFP) has been a serum biomarker to diagnose HCC; however, one large-scaled study showed that the sensitivity and specificity of AFP were 61% and 81% at a cutoff of 20 ng/mL and 22% and 100% at a cutoff of 200 ng/mL respectively, which were considered clinically suboptimal [3]. Notably, the value of AFP as a prognostic predictor has been investigated by different study groups, but clinical utility of AFP is still a subject of heated debate [4,5]. Most studies investigated the prognostic ability of AFP among HCC patients receiving specific therapies on a retrospective nature, and several important confounding factors such as tumor burden, severity of cirrhosis, vascular invasion and performance status were not comprehensively adjusted [6,7]. To the best of our knowledge, there was no prospective study focusing on the prognostic ability of AFP for unselected HCC patients.

Different cutoff values of AFP are used for different clinical settings. AFP level of 20 ng/mL or even lower were used for HCC patients undergoing surgical resection [8]. Other investigators chose AFP level of 200 or 1000 ng/mL to examine the prognostic ability of AFP in HCC patients undergoing liver transplantation [9]. Alternatively, AFP level of 400 ng/mL is included in two HCC staging systems, the Cancer of the Liver Italian Program (CLIP) and the Taipei Integrated Scoring (TIS) system, to predict long-term outcome of unselected HCC patients [10,11]. In addition to fixed AFP levels of 20, 200, 400 and 1000 ng/mL, other researchers have used median level of AFP of enrolled patients as the cutoff to examine the prognostic effect of AFP on long-term survival [12]. However, there was no study comparing the prognostic ability between different cutoffs thoroughly. In this study, we have analyzed the distribution and associated factors of different AFP levels in patients with HCC. In addition, in order to select the best cutoff of AFP for HCC patients after controlling potential confounders, we have analyzed the prognostic ability of 4 different cutoffs of AFP by using a propensity model; the adjusted hazard ratios were calculated in a large patient cohort.

## Patients and Methods

### Patients

A prospective database of patients with HCC collected during an 11-year period from 2002 to 2012 at Taipei Veterans General Hospital, Taiwan, formed the basis of this study. A total of 2579 treatment-naïve HCC patients were enrolled. The baseline information, including patient characteristics, causes of chronic liver disease, severity of cirrhosis, serum biochemistries, performance status and cancer stages, was recorded when the diagnosis was made. This study has been approved by the institutional review board of Taipei Veterans General Hospital and complies with the standards of the Declaration of Helsinki and current ethical guidelines. Waiver of consent was obtained, and patient records/information was anonymized and de-identified prior to analysis. Part of the study patients had been enrolled in our previous reports [11,13].

### Diagnosis and Definitions

The diagnosis of HCC was based on the findings of typical radiological characteristics in at least two imaging modalities including ultrasound, hepatic arterial angiography, magnetic resonance (MR) imaging and contrast-enhanced dynamic computed tomography (CT), or histologically confirmed, or by a single positive imaging technique accompanied with serum AFP level >400 ng/mL [14,15]. Hepatitis C virus (HCV) infection was diagnosed if patients were seropositive for antibody against HCV (anti-HCV) by a second-generation enzyme immunoassay (Abbott Laboratories). Hepatitis B virus (HBV) infection was diagnosed if hepatitis B surface antigen (HBsAg) was found serologically (RIA kits, Abbott Laboratories). Alcoholism was diagnosed in patients with daily consumption of at least 40 g of alcohol for 5 years or more [16]. Ascites defined as free peritoneal fluid was confirmed by abdominal sonography, MR or CT imaging [12]. Performance status was determined when HCC was diagnosed according to the Eastern Cooperative Oncology Group (ECOG) criteria [17]. Vascular invasion by tumor thrombus was confirmed by MR or CT imaging. The serum AFP level was measured by using a chemiluminescent immunoassay (Abbott Laboratories). The estimated glomerular filtration rate (eGFR) was calculated by using the modification of diet in renal disease (MDRD) formula [18]. Total tumor volume was calculated according to mathematical equations as described in our previous study [11].

### Treatment

Transplantation, surgical resection and percutaneous ablation therapy (acetic acid or ethanol injection and radiofrequency ablation) were collectively classified as curative treatments in this study. Transarterial chemoembolization (TACE), systemic chemotherapy, targeted therapy, and best supportive care were categorized in the group of non-curative treatments. Post-treatment follow-up including contrast-enhanced imaging studies, measurement of serum biochemistries in our hospital was performed every 3 months.

### Propensity score analysis

To compare the overall survival between HCC patients divided by cutoffs of AFP in a prospectively enrolled cohort, a propensity score model and greedy nearest neighbor matching without replacement were used to reduce potential biases in survival analysis [19,20]. Possible variables associated with long-term survival of HCC patients, including age, sex, etiologies of chronic liver disease, tumor burden, severity of cirrhosis, performance status, vascular invasion, renal function, curative treatments and the Barcelona-Clinic Liver Cancer (BCLC) stage were included comprehensively for propensity score generation. With these variables, a logistic regression was applied to calculate a continuous propensity score from 0 to 1. One-to-one matches between patients with AFP higher and lower than cutoffs were introduced into the subsequent analysis.

### Statistical Methods

For prognostic predictor analysis, continuous variables were split by the median or clinically meaningful values and treated as dichotomous covariates. Chi-squared test was performed for split variables among patients with different AFP levels. The comparison of survival distribution was performed by the Kaplan-Meier method with a log-rank test. Adjusted hazard ratios of high AFP were calculated by using the Cox proportional hazards model. A p value was considered statistically significant when it was less than 0.05. All statistical analyses were conducted with the SAS 9.0. (SAS institute, North Carolina)

## Results

### Characteristics of all patients

**Fig 1:**
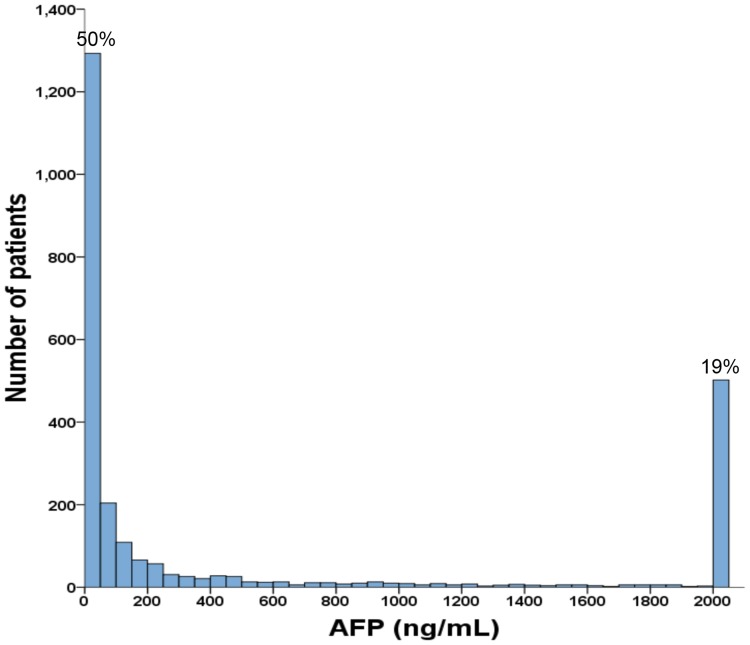
This histogram shows the distribution of AFP level of all patients in this study. Fifty percent of the study patients had a baseline AFP level less than 50 ng/mL and 19% of patients had a baseline AFP level over 2000 ng/mL.

As shown in Table 1, the mean age of the study patients was 64 years, and 77% were male. The most frequent cause of chronic liver disease was hepatitis B (55%), followed by hepatitis C (31%) and alcoholism (18%). Approximately 58% of patients were classified as performance status 0, and 72% of patients were diagnosed as Child-Turcotte-Pugh (CTP) class A. The majority of patients had single tumor (60%) and a main tumor size of 3 cm or more (68%). Vascular invasion was found in 29% of patients and 24% of patients had diabetes mellitus when HCC was found. There were 6%, 22%, 13%, 44% and 15% of patients belonging to BCLC stage 0, A, B, C and D, respectively. A total of 1172 (45%) of patients received curative treatments as primary anti-cancer management. There were 38% of patients with an AFP level lower than 20 ng/mL (0.7 to 19.99 ng/mL), followed by patients with an AFP level between 20 and 200 ng/mL (26%) and patients with an AFP level of 1000 ng/mL or higher (24%). For 19% of patients with an AFP lever over 2000 ng/mL, they were grouped together in the Fig. 1.

**Table 1:** Baseline demographics of the study patients.

Number of patients	2579
Age (years, mean ± SD [median])	64 ± 13 (65)
Male/female (%)	77/23
Etiology of cirrhosis (%)	
HBV	1421 (55)
HCV	798 (31)
HBV+HCV	116 (4)
Alcoholism	463 (18)
Serum biochemistry (mean ± SD [median])	
Albumin (g/dL)	3.7 ± 0.6 (3.7)
Bilirubin (mg/dL)	1.5 ± 2.7 (0.9)
Creatinine (mg/dL)	1.2 ± 1.0 (1)
INR of prothrombin time	1.1 ± 0.2 (1.1)
Sodium (mmol/L)	138 ± 4 (139)
Platelet (per μL)	172,732 ± 111,536 (152,000)
Estimated glomerular filtration rate (mL/min/1.73m^2^)	75 ± 32 (73.8)
α-fetoprotein (ng/mL, mean ± SD, [median])	24,407 ± 237,726 (49)
α-fetoprotein (ng/mL, %) <20	989 (38)
20–200	683 (26)
200–400	135 (5.2)
400–1000	161 (6.2)
≧1000	611 (24)
Performance status 0/1/2/3/4 (%)	58/18/12/8/4
CTP class A/B/C (%)	72/22/6
MELD score (median)	9.8 ± 4.2 (8.4)
Number and size of tumor (%)	
Single/multiple	60/40
< 3cm/≧3cm	32/68
Total tumor volume (cm^3^, median)	370 ± 725 (51)
Vascular invasion (%)	743 (29)
Ascites (%)	631 (24)
Diabetes mellitus (%)	612 (24)
Curative treatments (%)	1172 (45)
BCLC stage 0/A/B/C/D (%)	6/22/13/44/15

BCLC, Barcelona Clinic Liver Cancer; CTP, Child-Turcotte-Pugh; HBV, hepatitis B virus; HCV, hepatitis C virus; INR, international normalized ratio; MELD, model for end-stage liver disease; SD, standard deviation.

Curative treatments include surgical resection, percutaneous ablation and transplantation

### Associated factors of AFP levels

Distribution of baseline demographics in HCC patients according to AFP levels is shown in Table 2. In comparison with patients who had an AFP level lower than 20 ng/mL, patients with a higher AFP level had significantly poorer performance status, more advanced cirrhosis, and larger tumor burden (all p<0.0001). Patients with a higher AFP level also more often had hepatitis B, vascular invasion and ascites (all p<0.05). For patients with a higher baseline AFP, they were less likely to receive curative treatments as primary anti-cancer management (p<0.0001). The comparison across HCC patients with different AFP levels also showed significantly different distribution regarding age (p = 0.003), gender (p<0.0001), and hepatitis C (p<0.0001), but there was no specific trend in these 3 variables. The prevalence of alcoholism and diabetes mellitus between patients with different AFP levels were similar (both p>0.05).

**Table 2:** Comparison of HCC patients according to α-fetoprotein levels.

α-fetoprotein (ng/mL)						
	0–20 (n = 989)	20–200 (n = 683)	200–400 (n = 135)	400–1000 (n = 161)	≧1000 (n = 611)	p
Age ≧65 year (%)	55	51	55	46	45	0.003
Male (%)	82	72	73	75	76	<0.0001
HBV (%)	51	55	56	60	61	0.003
HCV (%)	29	37	31	38	26	<0.0001
Alcoholism (%)	16	17	18	18	22	0.0968
Serum biochemistry (%)						
Albumin ≧3.7g/dL	61	51	56	48	46	<0.0001
Bilirubin ≧0.9mg/dL	44	53	51	53	60	<0.0001
eGFR ≧60 mL/min/1.73m^2^	70	76	79	80	73	0.0036
INR of PT ≧1.05	43	55	64	58	60	<0.0001
Performance status 0 (%)	65	63	59	56	40	<0.0001
CTP classification A (%)	79	74	73	71	60	<0.0001
Tumor size ≧3cm (%)	60	58	60	70	92	<0.0001
Multiple tumor (%)	33	43	39	39	47	<0.0001
Tumor volume ≧51cm^3^	38	40	47	51	82	<0.0001
Vascular invasion (%)	15	22	32	33	58	<0.0001
Ascites (%)	20	20	21	29	37	<0.0001
Diabetes mellitus (%)	27	23	22	22	21	0.0708
Curative treatments (%)	56	48	46	39	26	<0.0001

eGFR, estimated glomerular filtration rate; PT, prothrombin time

### Survival comparison between HCC patients with different baseline AFP levels

**Fig 2:**
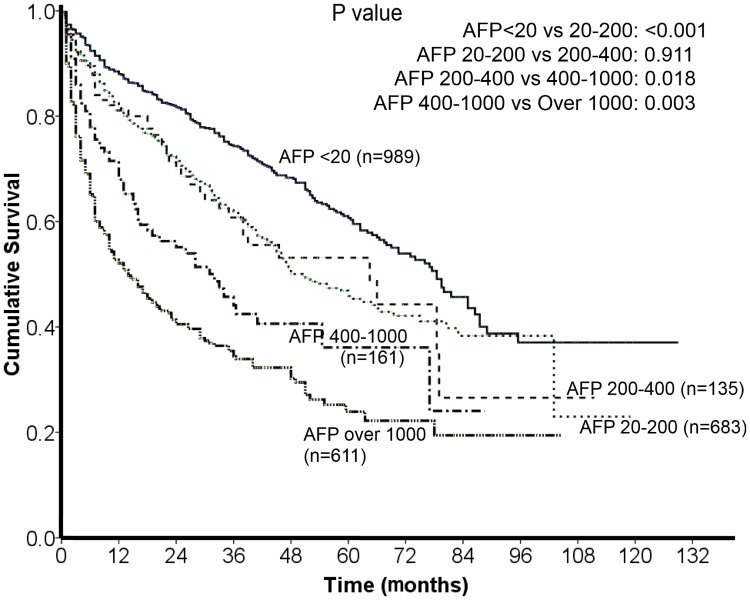
Comparison of survival distribution of patients with different AFP levels. There was a statistically significant difference across all groups of patients except for patients with AFP of 20 to 200 ng/mL and patients with AFP of 200 to 400 ng/mL.

The comparison of long-term survival between patients with different AFP levels is given in Fig. 2. During a mean follow-up period of 27° 26 months, patients with a higher AFP level had poorer long-term survival. Pairwise comparisons showed significant difference of long-term survival between each sequential group except for patients with an AFP level between 20 to 200 ng/mL and patients with an AFP level between 200 to 400 ng/mL (p = 0.911).

### Characteristics of patients selected in the propensity model

By using the propensity model, 777, 508, 396 and 377 pairs of baseline-matched HCC patients were selected according to 4 different cutoff values of AFP, respectively (Table 3). Among matched patients in these 4 subsets of patients, there were no significant differences in age, sex, etiology of chronic liver disease, severity of cirrhosis, renal function, tumor burden, performance status, ascites, vascular invasion, diabetes mellitus, treatment modality and the classification of BCLC system (all p>0.05).

**Table 3:** Comparison of baseline demographics in HCC patients stratified by 4 α-fetoprotein cutoff values in the propensity model.

Variables	N = 777 in each group		N = 508 in each group		N = 396 in each group		N = 377 in each group	
	<20/≧20 ng/mL	p	<200/≧200 ng/mL	p	<400/≧400 ng/mL	p	<1000/≧1000 ng/mL	p
Age ≧65 year (%)	53/52	0.6844	51/50	0.7065	50/49	0.6698	53/49	0.2745
Male (%)	79/80	0.6174	75/76	0.8273	78/76	0.6734	78/75	0.2299
HBV (%)	52/54	0.3341	55/57	0.3431	56/57	0.6673	57/57	1
HCV (%)	32/32	0.9132	28/31	0.2419	28/31	0.391	29/27	0.4166
Alcoholism (%)	16/18	0.1767	20/19	0.6346	21/17	0.1509	18/21	0.1679
CTP class A (%)	77/78	0.7616	68/71	0.3055	67/69	0.5417	66/67	0.7584
Ascites (%)	21/19	0.3392	28/25	0.2535	32/27	0.1375	34/30	0.31
eGFR ≧60 mL/min/1.73m^2^	71/75	0.1704	73/75	0.4755	75/74	0.9349	73/72	0.8705
Tumor volume ≧51cm^3^	39/43	0.1486	58/55	0.2821	63/63	0.9419	70/74	0.2916
Performance status 0 (%)	64/62	0.2695	56/56	1	48/47	0.7759	50/49	0.6621
Vascular invasion (%)	17/19	0.1651	34/29	0.0801	39/37	0.6083	44/44	0.9415
Diabetes mellitus (%)	26/23	0.1411	23/23	0.8229	23/23	1	24/24	0.8643
BCLC 0/A/B/C/D (%)	(8/26/15/39/12)/(8/25/12/44/10)	0.1408	(5/16/14/47/18)/(5/20/15/47/14)	0.2003	(4/14/10/51/21)/(2/17/14/52/15)	0.0594	(1/10/10/58/21)/(1/11/16/52/20)	0.1602
Curative treatments (%)	54/51	0.1701	41/40	0.8985	36/37	0.8248	35/31	0.2788

### Survival analysis of HCC patients in propensity model

**Fig 3:**
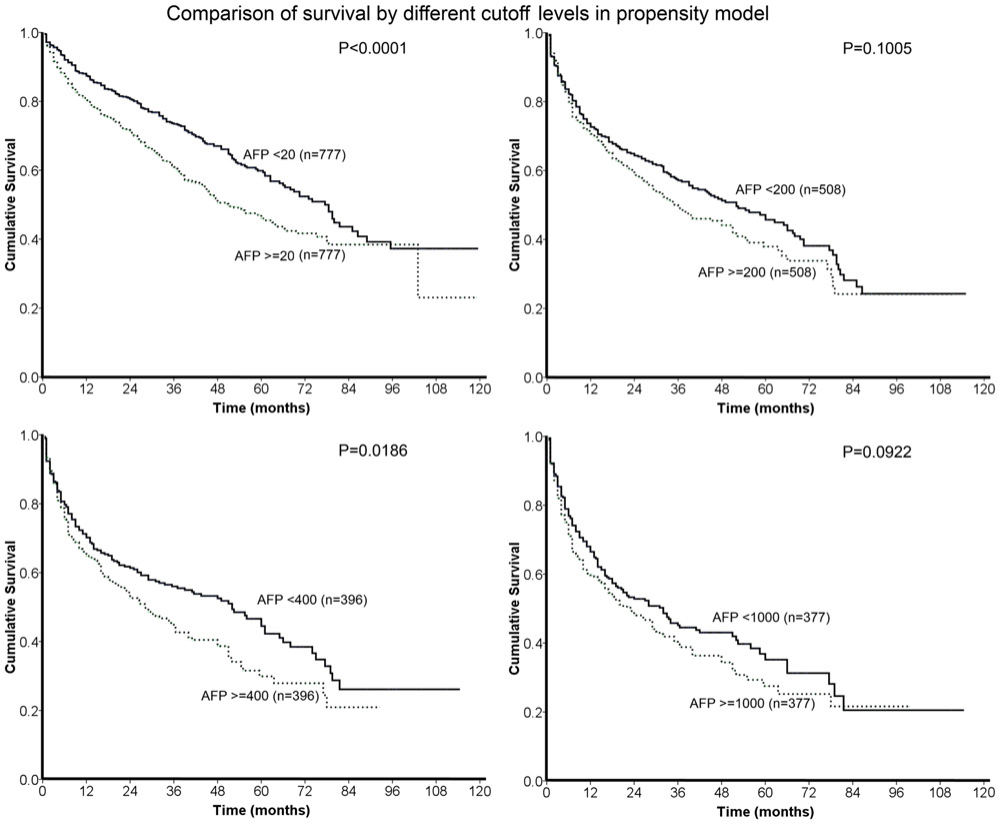
In the propensity model with 4 different cutoffs, AFP levels of 20 and 400 ng/mL, but not 200 and 1000 ng/mL, significantly differentiated long-term survival of HCC patients.

The comparisons of long-term survival between patients in the propensity model were showed in Fig. 3. There was no significant difference of overall survival between 2 groups of patients divided by AFP levels of 200 and 1000 ng/mL (both p>0.05). Patients with an AFP level ≧20 ng/mL had poorer long-term outcome than patients with an AFP level <20 ng/mL (p<0.0001); patients with an AFP level ≧400 ng/mL had shorter survival compared to patients with an AFP level <400 ng/mL (p = 0.0186). After adjusted by sex, gender, etiologies of chronic liver disease, severity of cirrhosis, renal function, tumor burden, performance status and vascular invasion, the hazard ratios of significant variables were shown in Table 4. For patients selected in the propensity model divided by AFP level of 20 ng/mL, AFP ≧20 ng/mL had an adjusted hazard ratio of 1.545 (95% confidence interval [CI]: 1.3–1.838, p<0.0001), and for patients selected in the propensity model divided by AFP level of 400 ng/mL, the adjusted hazard ratio of AFP ≧400 ng/mL was 1.471 (95% CI: 1.178–1.837, p = 0.0007). In addition, ascites, CTP classification, renal function, performance status, total tumor volume and vascular invasion were found significant on long-term survival prediction (all p<0.05).

**Table 4:** Independent prognostic predictors of HCC patients in the propensity model divided by α-fetoprotein levels of 20 and 400 ng/mL.

	Hazard ratio	95% confidence interval	p
All patients in propensity model divided by AFP level of 20 ng/mL (n = 1554)			
α-fetoprotein ≧20 ng/mL	1.545	1.3–1.838	<0.0001
Ascites	1.368	1.045–1.791	0.0225
CTP class B or C	2.02	1.58–2.583	<0.0001
MDRD <60 mL/min/1.73m^2^	1.335	1.101–1.615	0.0032
Performance status ≧1	1.702	1.372–2.11	<0.0001
Total tumor volume ≧51 cm^3^	1.761	1.451–2.138	<0.0001
Vascular invasion	2.192	1.749–2.746	<0.0001
All patients in propensity model divided by AFP level of 400 ng/mL (n = 792)			
α-fetoprotein ≧400 ng/mL	1.471	1.178–1.837	0.0007
Ascites	1.468	1.077–2	0.0151
CTP class B or C	1.919	1.405–2.621	<0.0001
Performance status ≧1	1.702	1.292–2.243	0.0002
Total tumor volume ≧51 cm^3^	1.614	1.236–2.107	0.0004
Vascular invasion	1.982	1.53–2.567	<0.0001

Adjusted hazard ratios were calculated by Cox proportional hazard model.

## Discussion

The expression of AFP in liver cancer is associated with cell proliferation, angiogenesis, and increased resistance of cells against tumor necrosis factor-related apoptosis [21,22]. Also, a higher AFP level is related to a more aggressive cancer phenotype and linked with hepatic cancer cells that have stem/progenitor features [23,24]. The clinical role of AFP in HCC has been studied by several independent research groups. However, only very few studies systemically investigated the prognostic impact of AFP levels on HCC. In addition, most studies focused on AFP levels in HCC patients undergoing a specific treatment in a retrospective dataset. In this study, the prognostic effects of 4 commonly used AFP cutoff values were investigated in a large prospective cohort by using propensity score analysis. AFP levels of 20 and 400 ng/mL were determined as significant cutoff values to predict long-term survival in non-selected HCC patients. Not only the etiology of chronic liver disease, tumor burden, renal function, severity of cirrhosis, and treatment modality, we also incorporated baseline performance status to generate adjusted hazard ratios of these two AFP cutoff values which could quantify the prognostic power of AFP. Our results indicate that baseline AFP levels of 20 and 400 ng/mL are useful cutoffs to predict long-term survival of HCC patients across different cancer stages and treatment modalities.

In studies evaluating the prognostic ability of AFP, the cutoff values varied widely between 20 to 1000 ng/mL [6]. With our prospective patient cohort, we had large case number in each group of patients divided by 4 AFP cutoffs as 20, 200, 400 and 1000 ng/mL. Sufficient sample size provides clearly right-skewed distribution of AFP level and a strong foundation for subsequent propensity score analysis. There were 38% of HCC patients had an AFP level lower than the normal upper limit (20 ng/mL) when HCC was diagnosed. Notably, 70% of patients had an AFP level lower than the diagnosis criteria (400 ng/mL) proposed by the European Association for the Study of the Liver in 2001 [14]. The distribution of AFP in this cohort explains the incompetence of AFP as a diagnostic tool of HCC. Recently, with improved accuracy of imaging studies and more sophisticated surveillance programs for patients with chronic liver diseases, more patients were found with small HCC accompanied by low AFP level; this tendency could further reduce the sensitivity of AFP as a diagnostic marker.

AFP level is frequently associated with tumor burden in HCC. In our study, larger main tumor size and total tumor volume well correlated with higher AFP level. However, number of tumor(s) in HCC patients was not associated with AFP level; this feature is consistent with the result of another independent study [25]. Also, patients with a higher AFP level also presented with higher prevalence of ascites and more advanced CTP class. Importantly, patients with a high AFP level had significantly worse performance status. This finding can be explained by the fact that patients with high AFP levels frequently have advanced HCC which is subsequently associated with poor performance status [17]. Considering the powerful influence of baseline performance status on overall survival in HCC patients, our prospective patient cohort provides more accurate adjusted hazard ratio of higher AFP level in comparison to the studies using a retrospective dataset [6,17].

The comparison between 5 groups of patients with different AFP levels shows significant difference of long-term survival between each comparison except for patients with AFP of 20–200 ng/mL and patients with AFP of 200–400 ng/mL. This finding implies that HCC patients with an AFP level within normal limit (20 ng/mL) had better survival than patients with an elevated AFP level, and patients with an AFP level ≧1000 ng/mL had the worst outcome. Because higher AFP levels are frequently accompanied by advanced HCC and poor performance status, a propensity model was used to reduce the confounding effect of other prognostic variables. Comparable compositions of patients with AFP levels higher or lower than cutoff values were selected in the propensity score analysis. Comparisons of long-term survival in these 4 groups showed that after other prognostic variables were adjusted, AFP levels of 20 and 400 ng/mL had significantly differential power in HCC patents in terms of survival prediction. On the other hand, AFP cutoff levels of 200 and 1000 ng/mL had no significant predictive role for long-term prognosis. This finding demonstrates that AFP≧1000 ng/mL should not be considered a useful prognostic predictor after other important variables such as tumor burden, severity of cirrhosis, treatment modality and performance status were adjusted. According to our results, the insignificant predictive ability of AFP level at 200 ng/mL could be resulted from similar distributions of survival between patients with AFP of 20–200 ng/mL and 200–400 ng/mL. Notably, both AFP cutoff levels of 20 and 400 ng/mL are used frequently in daily practice; 20 ng/mL is the upper limit of normal AFP level and 400 ng/mL is the cutoff value used in both the CLIP and TIS staging systems [10,11]. Our results further provide strong support of the rationality of AFP cutoff value in these two HCC staging systems.

There are a few limitations of this study. Firstly, in this study, more than half of our patients had hepatitis B infection. This feature is distinctly different from countries where hepatitis C infection was the predominant etiologies of chronic liver disease. Secondly, some patients with hepatitis B or C and alcoholic hepatitis might have ongoing hepatitis with different disease severity, and this could partially influence AFP levels in HCC patients. Lastly, AFP level was reported to associate with the grade of cancer cell differentiation [26]. Because approximately 50% of patients in this study did not have pathological data, the interaction of AFP and tumor differentiation is not clear.

In conclusion, our results indicate that patients with a higher baseline AFP level are associated with larger tumor burden, more severe cirrhosis and poorer performance status. Patients with a higher AFP level have decreased long-term survival. After confounding factors were adjusted in propensity score model, baseline quantities of AFP≧20 and ≧400 ng/mL are significant predictors of poor prognosis in unselected HCC patients. Our findings provide evidence-based support for the usefulness of AFP cutoff values in survival prediction, and for the rationale of their inclusion in the CLIP and TIS systems in cancer staging.

## References

[1] El-Serag HB, Marrero JA, Rudolph L, Reddy KR (2008) Diagnosis and treatment of hepatocellular carcinoma. Gastroenterology 134:1752–1763. 10.1053/j.gastro.2008.02.090 18471552

[2] Ramirez AG, Weiss NS, Holden AE, Suarez L, Cooper SP, et al (2012) Incidence and risk factors for hepatocellular carcinoma in Texas Latinos: implications for prevention research. PLoS One 7:e35573 10.1371/journal.pone.0035573 22530052 PMC3329468

[3] Lok AS, Sterling RK, Everhart JE, Wright EC, Hoefs JC, et al (2010) Des-gamma-carboxy prothrombin and alpha-fetoprotein as biomarkers for the early detection of hepatocellular carcinoma. Gastroenterology 138:493–502. 10.1053/j.gastro.2009.10.031 19852963 PMC2819612

[4] Shim JH, Yoon DL, Han S, Lee YJ, Lee SG, et al (2012) Is serum alpha-fetoprotein useful for predicting recurrence and mortality specific to hepatocellular carcinoma after hepatectomy? A test based on propensity scores and competing risks analysis. Ann Surg Oncol 19:3687–3696. 10.1245/s10434-012-2416-1 22644512

[5] Toyoda H, Kumada T, Kaneoka Y, Osaki Y, Kimura T, et al (2008) Prognostic value of pretreatment levels of tumor markers for hepatocellular carcinoma on survival after curative treatment of patients with HCC. J Hepatol 49:223–232. 10.1016/j.jhep.2008.04.013 18571271

[6] Hakeem AR, Young RS, Marangoni G, Lodge JP, Prasad KR (2012) Systematic review: the prognostic role of alpha-fetoprotein following liver transplant for hepatocellular carcinoma. Aliment Pharmacol Ther 35:987–999. 10.1111/j.1365-2036.2012.05060.x 22429190

[7] Merani S, Majno P, Kneteman NM, Berney T, Morel P, et al (2011) The impact of waiting list alpha-fetoprotein changes on the outcome of liver transplant for hepatocellular carcinoma. J Hepatol 55:814–819. 10.1016/j.jhep.2010.12.040 21334400

[8] Blank S, Wang Q, Fiel MI, Luan W, Kim KW, et al (2014) Assessing Prognostic Significance of Preoperative Alpha-Fetoprotein in Hepatitis B-Associated Hepatocellular Carcinoma: Normal is not the New Normal. Ann Surg Oncol 21:986–994. 24232510 10.1245/s10434-013-3357-z

[9] Sotiropoulos GC, Lang H, Nadalin S, Neuhauser M, Molmenti EP, et al (2007) Liver transplantation for hepatocellular carcinoma: University Hospital Essen experience and metaanalysis of prognostic factors. J Am Coll Surg 205:661–675. 17964442 10.1016/j.jamcollsurg.2007.05.023

[10] The Cancer of the Liver Italian Program (CLIP) Investigators (2000) Prospective validation of the CLIP score: a new prognostic system for patients with cirrhosis and hepatocellular carcinoma. Hepatology 31:840–845. 10733537 10.1053/he.2000.5628

[11] Hsu CY, Huang YH, Hsia CY, Su CW, Lin HC, et al (2010) A new prognostic model for hepatocellular carcinoma based on total tumor volume: the Taipei Integrated Scoring System. J Hepatol 53:108–117. 10.1016/j.jhep.2010.01.038 20451283

[12] Hsu CY, Lee YH, Huang YH, Hsia CY, Su CW, et al (2013) Ascites in patients with hepatocellular carcinoma: prevalence, associated factors, prognostic impact, and staging strategy. Hepatol Int 7:188–198. 26201633 10.1007/s12072-011-9338-z

[13] Lee YH, Hsu CY, Huang YH, Su CW, Lin HC, et al (2012) Selecting a prognostic renal surrogate for patients with hepatocellular carcinoma undergoing transarterial chemoembolization. J Gastroenterol Hepatol 27:1581–1588. 10.1111/j.1440-1746.2012.07151.x 22497632

[14] Bruix J, Sherman M, Llovet JM, Beaugrand M, Lencioni R, et al (2001) Clinical management of hepatocellular carcinoma. Conclusions of the Barcelona-2000 EASL conference. European Association for the Study of the Liver. J Hepatol 35:421–430. 11592607 10.1016/s0168-8278(01)00130-1

[15] Bruix J, Sherman M (2005) Management of hepatocellular carcinoma. Hepatology 42:1208–1236. 16250051 10.1002/hep.20933

[16] Lee YH, Hsu CY, Hsia CY, Huang YH, Su CW, et al (2013) Alcoholism worsens the survival of patients with hepatitis B virus and C virus-related hepatocellular carcinoma. Hepatol Int 7:645–654. 26201797 10.1007/s12072-012-9375-2

[17] Hsu CY, Lee YH, Hsia CY, Huang YH, Su CW, et al (2013) Performance status in patients with hepatocellular carcinoma: determinants, prognostic impact, and ability to improve the Barcelona Clinic Liver Cancer system. Hepatology 57:112–119. 10.1002/hep.25950 22806819

[18] Gonwa TA, Jennings L, Mai ML, Stark PC, Levey AS, et al (2004) Estimation of glomerular filtration rates before and after orthotopic liver transplantation: evaluation of current equations. Liver Transpl 10:301–309. 14762871 10.1002/lt.20017

[19] Austin PC (2014) A comparison of 12 algorithms for matching on the propensity score. Stat Med 33:1057–1069. 10.1002/sim.6004 24123228 PMC4285163

[20] Hsu CY, Lee YH, Hsia CY, Huang YH, Su CW, et al (2013) Performance status enhances the selection of treatment for patients with hepatocellular carcinoma within the milan criteria. Ann Surg Oncol 20:2035–2042. 10.1245/s10434-012-2847-8 23306955

[21] Mitsuhashi N, Kobayashi S, Doki T, Kimura F, Shimizu H, et al (2008) Clinical significance of alpha-fetoprotein: involvement in proliferation, angiogenesis, and apoptosis of hepatocellular carcinoma. J Gastroenterol Hepatol 23:e189–197. 10.1111/j.1440-1746.2008.05340.x 18466288

[22] Yang X, Zhang Y, Zhang L, Zhang L, Mao J (2008) Silencing alpha-fetoprotein expression induces growth arrest and apoptosis in human hepatocellular cancer cell. Cancer Lett 271:281–293. 10.1016/j.canlet.2008.06.017 18657899

[23] Tangkijvanich P, Anukulkarnkusol N, Suwangool P, Lertmaharit S, Hanvivatvong O, et al (2000) Clinical characteristics and prognosis of hepatocellular carcinoma: analysis based on serum alpha-fetoprotein levels. J Clin Gastroenterol 31:302–308. 11129271 10.1097/00004836-200012000-00007

[24] Yamashita T, Ji J, Budhu A, Forgues M, Yang W, et al (2009) EpCAM-positive hepatocellular carcinoma cells are tumor-initiating cells with stem/progenitor cell features. Gastroenterology 136:1012–1024. 10.1053/j.gastro.2008.12.004 19150350 PMC2828822

[25] Wang Y, Chen Y, Ge N, Zhang L, Xie X, et al (2012) Prognostic significance of alpha-fetoprotein status in the outcome of hepatocellular carcinoma after treatment of transarterial chemoembolization. Ann Surg Oncol 19:3540–3546. 10.1245/s10434-012-2368-5 22532305

[26] Fujiki M, Takada Y, Ogura Y, Oike F, Kaido T, et al (2009) Significance of des-gamma-carboxy prothrombin in selection criteria for living donor liver transplantation for hepatocellular carcinoma. Am J Transplant 9:2362–2371. 10.1111/j.1600-6143.2009.02783.x 19656125

